# Interaction between Diethyldithiocarbamate and Cu(II) on Gold in Non-Cyanide Wastewater

**DOI:** 10.3390/s17112628

**Published:** 2017-11-15

**Authors:** Nguyễn Hoàng Ly, Thanh Danh Nguyen, Kyung-Duk Zoh, Sang-Woo Joo

**Affiliations:** 1 Department of Chemistry, Soongsil University, Seoul 156-743, Korea; nguyenhoangly2007@gmail.com (N.H.L.); nguyenthanhdanh1802@gmail.com (T.D.N.); 2 Department of Information Communication, Materials, Chemistry Convergence Technology, Soongsil University, Seoul 156-743, Korea; 3 Department of Environmental Health Sciences, School of Public Health, Seoul National University, Seoul 08826, Korea

**Keywords:** surface-enhanced Raman spectroscopy, plasmonic gold nanoparticles, diethyldithiocarbamate, industrial electroplating wastewater, cyanide removal

## Abstract

A surface-enhanced Raman scattering (SERS) detection method for environmental copper ions (Cu^2+^) was developed according to the vibrational spectral change of diethyldithiocarbamate (DDTC) on gold nanoparticles (AuNPs). The ultraviolet-visible (UV-Vis) absorption spectra indicated that DDTC formed a complex with Cu^2+^, showing a prominent peak at ~450 nm. We found Raman spectral changes in DDTC from ~1490 cm^−1^ to ~1504 cm^−1^ on AuNPs at a high concentration of Cu^2+^ above 1 μM. The other ions of Zn^2+^, Pb^2+^, Ni^2+^, NH_4_^+^, Mn^2+^, Mg^2+^, K^+^, Hg^2+^, Fe^2+^, Fe^3+^, Cr^3+^, Co^2+^, Cd^2+^, and Ca^2+^ did not produce such spectral changes, even after they reacted with DDTC. The electroplating industrial wastewater samples were tested under the interference of highly concentrated ions of Fe^3+^, Ni^2+^, and Zn^2+^. The Raman spectroscopy-based quantification of Cu^2+^ ions was able to be achieved for the wastewater after treatment with alkaline chlorination, whereas the cyanide-containing water did not show any spectral changes, due to the complexation of the cyanide with the Cu^2+^ ions. A micromolar range detection limit of Cu^2+^ ions could be achieved by analyzing the Raman spectra of DDTC in the cyanide-removed water.

## 1. Introduction

The resonant plasmonic enhancement of the local electric field on noble metal substrates could provide a highly sensitive platform for achieving single-molecule label-free detection [1]. SERS has been applied for detecting traces of organic compounds adsorbed on metal substrates [2]. A localized surface plasmon band of AuNPs can be utilized as a platform for studying chemical and biological reactions [3]. Plasmonic nanoparticles have been introduced to study heavy metal pollutants [4,5,6]. Detailed molecular interactions between organic compounds and metal atoms can be estimated with a combination of quantum-mechanical density functional theory (DFT) calculations [7,8].

Developments in sensing, identifying, and detecting heavy metal ions in aqueous solutions could contribute to the advanced treatment of wastewater [9]. The treatment of heavy metal ions in wastewater has been a significant challenge for environmental scientists [10]. However, because of the high toxicity of cyanide (CN) species, electroplating wastewater can be categorized into two kinds of wastewater: cyanide and non-cyanide [11]. Chlorination in highly alkaline conditions can be an efficient method for the reduction of cyanide ions in wastewater [12,13,14,15]. 

Diethyldithiocarbamate (DDTC) has been known to bind with metal complexes [16,17,18]. Among the various metal ions, Cu^2+^ has been found to interact strongly with DDTC [19,20,21,22]. Plasmonic nanoparticle-mediated SERS has been employed to detect hazardous species, such as heavy metal ions, in environmental samples [23]. The SERS spectrum of the DDTC-Cu^2+^ complex has recently been reported in a combination of quantum mechanical calculations [24]. 

The development of new, convenient methods for the spectroscopic characterization of contaminants in water effluent is still of significant importance in environmental fields [25,26]. Despite various methods for detecting ionic species [27] in aqueous solutions, there has been no report of a Raman spectroscopy-based analytical approach to DDTC-correlated copper ion quantification in real industrial wastewater samples. In this work, we found that SERS could be applied for detecting Cu^2+^ ions with the micromolar sensitivity in non-cyanide wastewater samples. In the case of cyanide presence in wastewater, samples need to be treated with alkaline chlorination before Cu^2+^ detection. The potential application of this research is to develop a quick and easy spectroscopic tool to estimate the toxic amounts of harmful chemicals in wastewater. Our work may also be used to detect the presence of cyanide ions in wastewater samples before and after alkaline chlorination.

## 2. Materials and Methods

### 2.1. Materials and Preparation of AuNPs and DDTC-Metal Complex

Sodium diethyldithiocarbamate trihydrate ((C_2_H_5_)_2_NCSSNa·3H_2_O) and the metal ionic substances were purchased from Sigma Aldrich (St. Louis, MO, USA). AuNPs were prepared by the previous method [28]. Our quantification methods for Cu^2+^ ions were based on our recent investigation [29]. 

We prepared AuNPs using a citrate reduction method. First, a triple-distilled water solution of hydrogen tetrachloroaurate trihydrate was mixed, stirred, and heated to the boiling point of water. Subsequently, sodium citrate was quickly added to this mixture and continuously stirred while the mixture was boiling. Finally, the level of the reaction solution was always kept at the beginning levels for 1 h by adding triple-distilled water slowly and continuously. The AuNPs were obtained at about 20 nm in diameter according to the measurements by Otsuka ELZ-2 and high-resolution transmission electron microscope (TEM) (JEOL JEM-3100). 

To prepare the UV-Vis experiment with the DDTC-Cu^2+^ complex, all of DDTC (0.89 mM in triple-distilled water, 500.0 µL) and Cu^2+^ (1.0 mM in triple-distilled water, 50.0 µL) were put into a 2.0 mL Eppendorf tube as a first step. This mixture solution (pH = 7.0) was stirred and kept stable for 30 min at room temperature. In the second step, 450.0 µL of triple-distilled water was added into this mixture to obtain 1000.0 µL solution of DDTC-Cu^2+^ complex with 50.0 µM final concentration of Cu^2+^. After that, UV-Vis spectra of 1000.0 µL of DDTC-Cu^2+^ complex was recorded. Samples with other metal ions were prepared by substituting Cu^2+^. 

For the SERS experiment of DDTC-Cu^2+^ complex on AuNPs, in the first step, all of the DDTC (8.9 mM in triple-distilled water, 50.0 µL) and Cu^2+^ (10.0 mM in triple-distilled water, 5.0 µL) was put into a 2.0 mL Eppendorf tube. This mixture solution (pH = 7.0) was stirred and kept stable for 30 min at room temperature. In the second step, 445.0 µL of AuNP solution was added into this mixture to obtain a 500.0 µL solution of the DDTC-Cu^2+^ complex on AuNPs with a 100.0 µM final concentration of Cu^2+^. Then, SERS spectra of 500.0 µL of the DDTC-Cu^2+^ complex on AuNPs was recorded. Samples with other metal ions were prepared by substituting Cu^2+^.

### 2.2. Instrumentations and DFT Calculations

UV-Vis absorption spectral changes of the DDTC-metal complexes before and after applying to the AuNP colloidal solution were obtained with a 3220 PC spectrophotometer (Mecasys, Daejeon, Korea). Atomic percentages in wastewater was obtained using a NEXION 350 D ICP-MS spectrometer (Perkin-Elmer, Boston, MA, USA). DFT calculations [30] and potential energy distribution were performed using the previous literatures [31]. The Au_6_ cluster is one of the simplest models of gold atoms. It includes calculations to predict the energetic stabilities and intramolecular interactions of the adsorbates on Au surfaces. This model includes six gold atoms that can connect with each other to generate the triangular geometry needed to form gold clusters.

All Raman data were obtained by using a Raman microscope system RM 1000 spectrometer (Renishaw, Gloucestershire, UK) with a 632.8 nm HeNe excitation laser and a CCD camera. The SERS spectra were recorded by using spectroscopic glass tubes after the preparation of the Cu^2+^ detection samples. The integration time for SERS measurement was set up at 10 s per spectrum with a range of 200–3200 cm^−1^. Prior to performing SERS, the spectral positions were calibrated based on Si peak at 520 cm^−1^.

### 2.3. Preparation of Wastewater Samples and Removal of the Cyanide Species Using Alkaline Chlorination

The real water samples were obtained from the wastewater treatment center (Pusan, Korea). The cyanide reactions could be derived from the previous literature [32]. Samples “S1” and “S2” were obtained before and after the process of removing the cyanide species, respectively. 

## 3. Results and Discussion

### 3.1. Adsorption of DDTC-Cu^2+^ on AuNPs

**Scheme 1:**
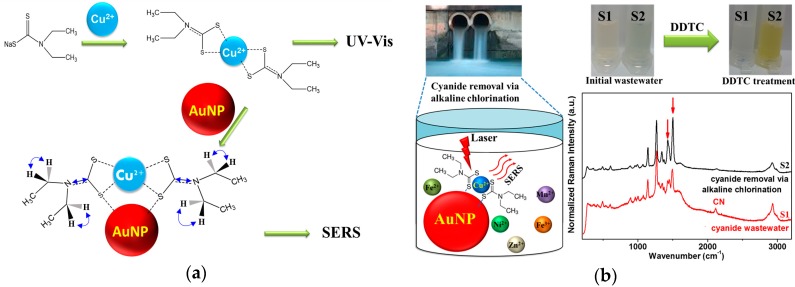
Schematic diagram of detection of Cu^2+^ ions with (**a**) a complex of DDTC and subsequent adsorption on AuNPs and (**b**) in electroplating wastewater after alkaline chlorination under the interference from the other ionic species.

Scheme 1 is a diagram of our detection of Cu^2+^ ions with a complex of DDTC and subsequent adsorption on AuNPs. As shown in Scheme 1a, our method would be able to detect Cu^2+^ ions not only using the UV-Vis method, but, more importantly, also based on the SERS tool, which could demonstrate a much higher selectivity and sensitivity capacity than the UV-Vis method. 

In general, our method could be promisingly applied to wastewater samples in both the presence of CN^−^ and the absence of CN^−^. Under wastewater conditions without CN^−^, the results indicate the ability for DDTC-Cu^2+^ complex detection via both the colorimetric indicator and the SERS tool (as shown in Scheme 1b with the S2 sample). Moreover, in the case of the presence of CN^−^ (the S1 sample), our approach may also be useful as a quick and simple method to identify the cyanide species in electroplating industrial water, since the copper cyanide complex cannot bind efficiently with DDTC, based on the results of UV-Vis and SERS in comparison with the S2 sample.

**Figure 1:**
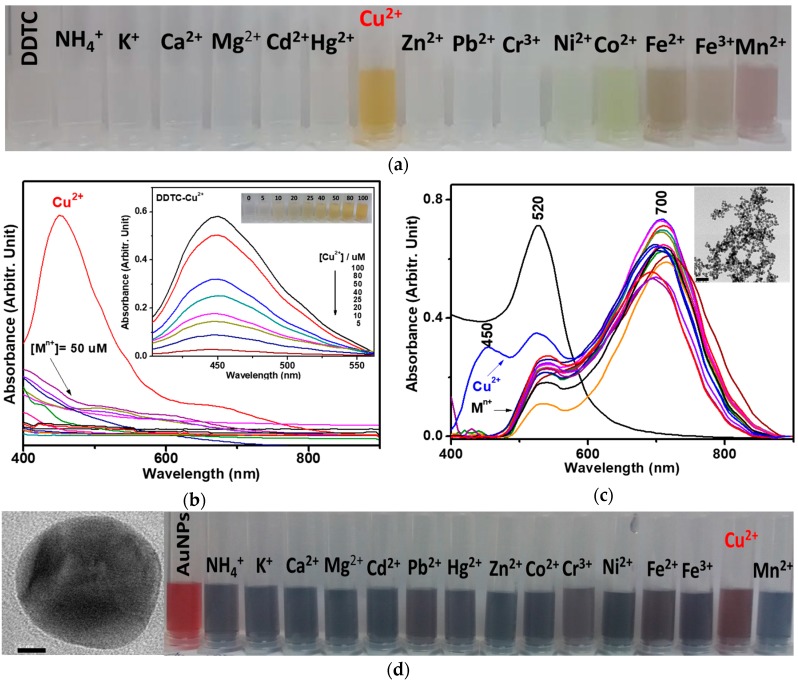
(**a**) Photo of DDTC and DDTC-metal complexes for NH_4_^+^, K^+^, Ca^2+^, Mg^2+^, Cd^2+^, Pb^2+^, Hg^2+^, Zn^2+^, Co^2+^, Cr^3+^, Ni^2+^, Fe^2+^, Fe^3+^, Cu^2+^, and Mn^2+^ ions. (**b**) UV-Vis absorption spectra of DDTC-metal complexes. The inset shows the absorption bands at ~450 nm and the photo of DDTC-Cu^2+^ complex corresponding to various concentrations of the Cu^2+^ ion. (**c**) UV-Vis absorption spectra of AuNPs and DDTC-metal complexes on AuNPs. The inset shows the TEM image of the AuNPs-DDTC-Cu^2+^ complex. The scale bar is 100 nm. (**d**) High-resolution image of AuNPs with a scale bar of 5 nm. Photo of AuNPs and DDTC-metal complexes on AuNPs.

Figure 1a shows a photo of DDTC-metal complexes. The Cu^2+^ ion exhibited a yellow color, which was supported by the UV-Vis absorption spectra of DDTC-metal complexes, as shown in Figure 1b. The inset in Figure 1b shows the UV-Vis absorption spectra, as well as a photo of DDTC-Cu^2+^ corresponding to various concentrations of the Cu^2+^ ion. The absorption bands of DDTC-Cu^2+^ exhibited prominent bands at ~450 nm, which is in different from other tested ions as previous reported [21]. The band at 520 nm is the spectrum of the pristine AuNPs just after synthesis, without any DDTC complexes. In Figure 1c, upon adsorption on AuNP surfaces, the plasmonic bands of initial AuNPs at 520 nm became considerably redshifted to ~700 nm, indicating the aggregation of AuNPs due to the strong binding of sulfur atoms in DDTC on Au. Although we found that the aggregation-induced color and UV-Vis spectral changes after adsorption upon AuNPs in Figure 1d did not depend on the metal ionic species, presumably due to the strong binding of the sulfur atoms in DDTC on Au, the strong binding of the Cu^2+^ ions with DDTC as shown in Figure 1a,b may change the minute adsorption characteristics on AuNPs. Despite the extensive aggregation of AuNPs, regardless of the complex types of DDTC with different ions as indicated in Figure 1d, SERS may exhibit minute spectral changes depending on the binding modes. As listed in Table 1, our DFT calculations predicted that the ν(N=C) mode at 1490–1520 cm^−1^ for DDTC could be sensitively changed on Au atoms, depending on the binding modes and the presence of Cu(II) ions. To find any different interfacial interactions, we performed Raman spectroscopy.

### 3.2. Raman Spectra of DDTC-Cu^2+^ on AuNPs

**Figure 2:**
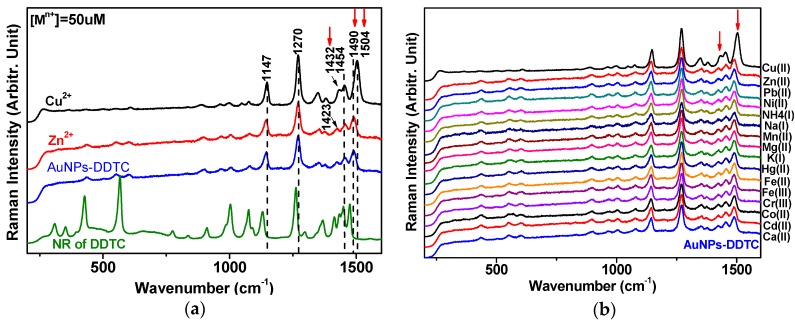
(**a**) Normal Raman (NR) for the solid state of DDTC and SERS spectra of DDTC, DDTC-Zn^2+^, and DDTC-Cu^2+^ on AuNPs. In the case of the DDTC-Cu^2+^ complex, the vibrational band at ~1490 cm^−1^ was prominently blueshifted to ~1504 cm^−1^, as marked in red arrows. (**b**) The SERS spectra of DDTC-metal complexes on AuNPs for Cu^2+^, Zn^2+^, Pb^2+^, Ni^2+^, NH_4_^+^, Na^+^, Mn^2+^, Mg^2+^, K^+^, Hg^2+^, Fe^2+^, Fe^3+^, Cr^3+^, Co^2+^, Cd^2+^, and Ca^2+^.

Figure 2a exhibits normal Raman (NR) for the solid state of DDTC, and the SERS spectra of DTTC, DDTC-Zn^2+^, and DDTC-Cu^2+^ on AuNPs. Our spectra appear consistent with that in the previous report [24]. Notably, the vibrational band at ~1490 cm^−1^ was prominently blueshifted to ~1504 cm^−1^, as marked in red arrows in the case of DDTC-Cu^2+^. Such spectral changes were not observed for the SERS spectra of DDTC-metal complexes on AuNPs, as illustrated in Figure 2b. The difference may be due to the exceptionally high binding energy of the Cu^2+^ ion to DDTC. To explain the spectral changes, the quantum mechanical DFT calculations were introduced to better assign the Raman peaks of DDTC with a complex of the metal ion in a free and adsorbed state on Au_6_ cluster atoms. An appropriate vibrational assignment is summarized in Table 1.

### 3.3. DFT Calculations of DDTC-Cu^2+^ on AuNPs

The DFT calculated spectra of DDTC and DDTC-Cu^2+^ on the Au_6_ cluster under the polarizable continuum model (PCM), appeared to match well with the SERS spectrum of the experimental Raman spectrum of DDTC. The bands at ~1423 and ~1490 cm^−1^ can be both ascribed to the vibrational modes of C=N stretching and CH_2_ bending modes. Of these, the stretching vibration of C=N is supposed to be more Raman-active dominant. These two bands, which are sensitive to the C=N stretching modes, can be expected to change considerably on AuNPs if the copper ion replaces the sulfur atom, leading to change in the relative Raman signals based on the present theoretical calculation. Although not shown here, the bond length of C=N of DDTC appeared to decrease from 1.39 to 1.34 Å after binding to Cu^2+^, resulting in increased vibrational frequencies.

### 3.4. Quantification of the Cu^2+^ Ion on the Basis of Raman Spectra

According to a previous report [33], the C=N stretching vibrational frequency should increase when the Schiff base is bound to Lewis acids such as H^+^ and BF_3_. Considering that the Cu^2+^ ion, as an electron pair receptor, could play the role of a Lewis acid, its binding to the nitrogen atom could increase the C=N stretching vibrational frequencies. A recent SERS study also indicated that the DDTC-based thiram and ziram may coordinate with a gold film as either a monodentate or a bidentate mode on Au [34].

**Figure 3:**
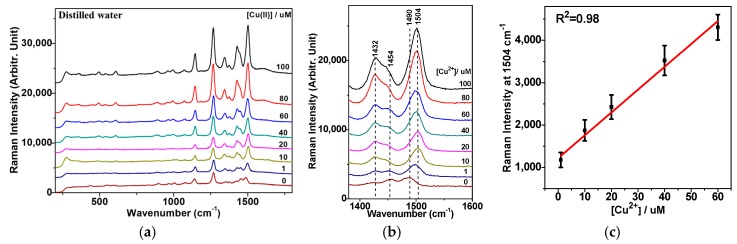
(**a**) Cu^2+^ concentration-dependent SERS spectra of DDTC on AuNPs in distilled water. (**b**) A magnified view of the spectral region from 1380 to 1600 cm^−1^. (**c**) Three independent measurements of Raman intensities of vibrational bands at ~1504 cm^−1^ were performed to provide the standard deviations and a linear fit for the concentration range between 1 and 60 μM.

Figure 3a shows the concentration-dependent SERS spectra of DDTC on AuNPs. The peak intensities at ~1504 cm^−1^ steadily increased, depending on the concentration of the Cu^2+^ ion, as magnified in Figure 3b. The calibration curve of the Raman peak intensities versus [Cu^2+^] is shown in Figure 3c.

### 3.5. Raman Spectroscopic Quantification of Cu^2+^ Ions in Electroplating Wastewater Samples

**Figure 4:**
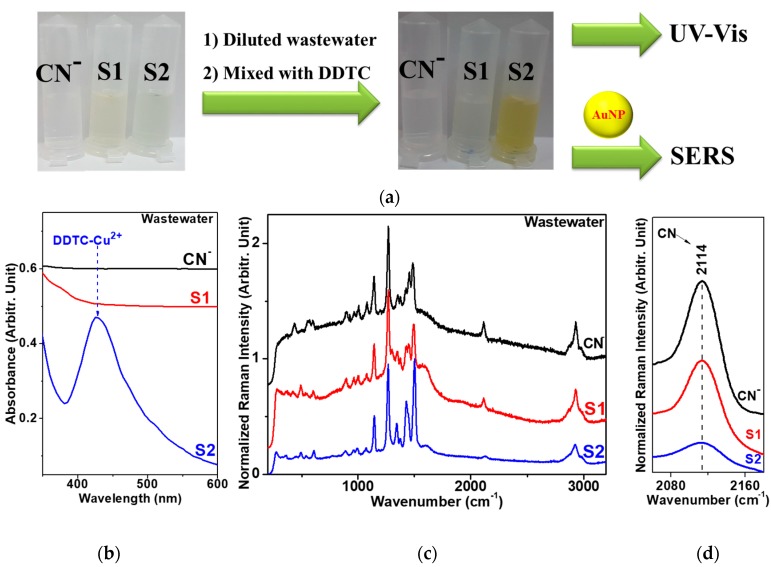
(**a**) Initial photo of the industrial wastewater samples: [CN] = 100 ppm, “S1” (cyanide-containing), and “S2” (after alkaline chlorination). Photo of wastewater samples after dilution and treatment with DDTC. (**b**) UV-Vis absorption spectra of [CN], “S1”, and “S2” after treatment with DDTC. (**c**) SERS spectra of [CN], “S1”, and “S2” on AuNPs after treatment with DDTC. (**d**) A magnified view of the C≡N triple bond stretching region at ~2114 cm^−1^.

Considering that alkaline chlorination can remove hazardous cyanide species from wastewater samples [12,13,14,15], we applied our method to detecting the presence of cyanide ions in real wastewater samples before and after alkaline chlorination. Table 2 shows the atomic percentages of various metal ionic species in electroplating wastewater samples. Figure 4 shows our spectroscopic results from the electroplating wastewater samples: standard solution of [CN] = 100 ppm, “S1” (cyanide-containing), and “S2” (after alkaline chlorination). Under our experimental conditions, UV-Vis absorption and SERS spectra of DDTC on AuNPs, depending on the concentration of Cu^2+^ for sample “S2” after alkaline chlorination, looked similar to those in the distilled water, whereas the cyanide-containing samples of the standard and “S1” did not exhibit such behaviors. The complex formation of the Cu^2+^ ion with the CN species may hamper the binding of Cu^2+^ and DDTC. In Figure 4b, the absorption band was weakened for the cyanide-containing wastewater samples. This interpretation could also be supported by the relatively weak CN intensity at ~2114 cm^−1^ [29] of the wastewater after the alkaline chlorination treatment of sample “S2”, as shown in Figure 4c.

**Figure 5:**
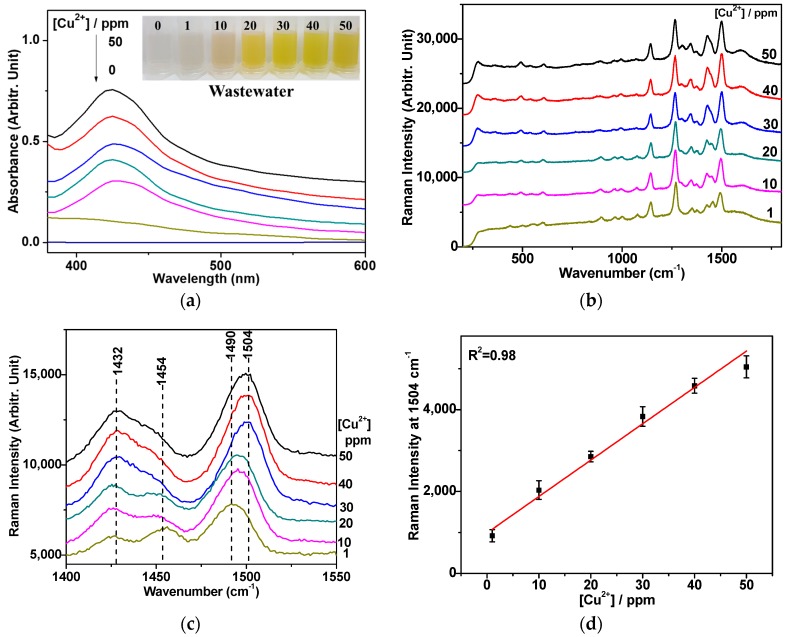
(**a**) UV-Vis absorption spectra of the DDTC-Cu^2+^ complex in wastewater with bands at ~450 nm, depending on the concentrations of the Cu^2+^ ion. The inset shows a photo of the DDTC-Cu^2+^ complex with [Cu^2+^] from 0 to 50 ppm in wastewater. (**b**) Cu^2+^ concentration-dependent SERS spectra of DDTC on AuNPs in wastewater samples after alkaline chlorination treatment. (**c**) A magnified view of the region from 1400 to 1550 cm^−1^. (**d**) Three independent measurements of Raman intensities at vibrational bands of ~1504 cm^−1^ were performed to provide the standard deviations and a linear fit for the concentration range between 1 and 50 ppm. The samples for the calibration curve were obtained by dilution of the initial wastewater (77.06 ppm Cu^2+^) with the DDTC-Cu^2+^ complex and the AuNP solution. The samples with the other Cu^2+^ concentrations could also made by changing the volumes of wastewater and DDTC-Cu^2+^ complex, accordingly.

**Figure 6:**
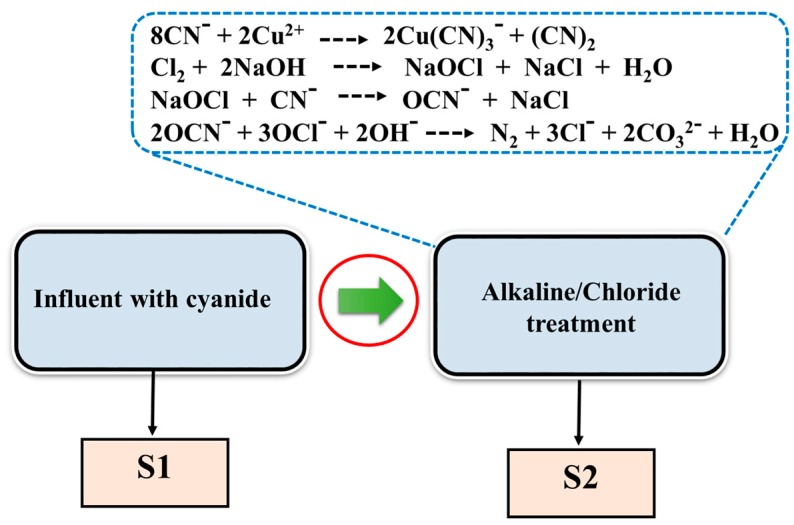
An alkaline chlorination process of the influent sample “S1” to remove the cyanide species in electroplating industrial wastewater to produce the non-cyanide wastewater in sample “S2”.

Figure 5 shows the concentration-dependent SERS spectra of DDTC on AuNPs in electroplating the wastewater sample “S2”. The peak intensities at ~1504 cm^−1^ steadily increased, depending on the concentration of the Cu^2+^ ion, similar to the case of the distilled water. The detection limit of the current SERS method was found to be around ten times lower than that of the colorimetric test under our experimental conditions. The lowest concentration of our SERS detection of Cu^2+^ in wastewater samples was around 1 ppm, which is lower than the Environmental Protection Agency permission level (1.3 ppm = ~20 μM) for drinkable water [29]. Figure 6 illustrates the alkaline chlorination process of the influent sample “S1” to remove the cyanide species in electroplating the industrial wastewater to produce non-cyanide wastewater “S2”. The following equations can be applied to treat the cyanide wastewater by alkaline chlorination.

8CN^−^ + 2Cu^2+^ → 2Cu(CN)_3_^−^ + (CN)_2_ (Stoichiometry cyanide reaction with Cu^2+^)

Cl_2_ + 2NaOH → NaOCl + NaCl + H_2_O (alkaline chlorination)

NaOCl + CN^−^ → OCN^−^ + NaCl (Destruction of CN^−^)

2OCN^−^ + 3OCl^−^ + 2OH^−^ → N_2_ + 3Cl^−^ + 2CO_3_^2−^ + H_2_O (Destruction of OCN^−^ and OCl^−^)

Alkaline chlorination can be divided into two steps. (1) A highly alkaline condition (pH > 10) will suppress the generation of gaseous HCN to maintain the free cyanide ions in wastewater (workers should be cautious about exposure to the gas in the atmosphere). A chlorine gas injection at least seven times higher than that of cyanide will yield cyanate (OCN^−^), avoiding the formation of the metal cyanide complexes and the other chlorine adducts, including cyanogen chloride (CNCl). (2) The cyanide adducts, such as OCN^−^, can be destroyed by lowering the pH to 8.5 to decompose them to CO_2_ and N_2_.

## 4. Conclusions

Our study showed that a facile detection method for Cu^2+^ ions in the DDTC-metal complexes could be achieved by monitoring specific marker bands in the SERS spectra. The SERS bands at ~1504 cm^−1^ increased after the introduction of DDTC-Cu^2+^ complexes on AuNPs. This could be interpreted as the conformation of the complex that would have different orientations on Au as supported by DFT calculations. The other ions of Ni^2+^, Fe^2+^, Co^2+^, Mn^2+^, Zn^2+^, Pb^2+^, Mg^2+^, Cd^2+^, Ca^2+^, Hg^2+^, NH_4_^+^, Cr^3+^, Fe^3+^, and K^+^ did not exhibit such spectral behaviors. The UV-Vis absorption and a colorimetric method were also introduced to check the [Cu^2+^]-induced spectroscopic changes. Our method can be successfully applied to real electroplating wastewater samples. After removal of the CN species via alkaline chlorination, the DDTC spectral features could be correlated with the concentration of Cu^2+^.

## Figures and Tables

**Table 1:** Spectral data and vibrational assignments for DDTC and DDTC-Cu^2+^ on Au.

NR of DDTC	^a^ DFT DDTC-Au_6_	SERS on Au	^a^ DFT Cu(DDTC)_2_-Au_6_	SERS on Au	^b^ Assignments Based on PED Calculations
---	---	---	268	268	β(C–N–C) + β(N–C–S)
350	329	---	345	367	ν(Cu–S) + β(S–C–S)
426	415	435	407	434	β(C–C–N) + ν(S–C) + γ(N–S–S–C)
567	523	552	523	547	ν(S–C) + γ(N–S–S–C) + β(C–N–C)
775	756	---	756	---	ν(N=C)(CH_2_)
835	849	---	841	---	δ(H–C–C–N)
910	935	902	942	885	ν(C–C)
1003	997	1005	997	998	ν(N=C)(CH_2_) + ν(S–C) + ν(C–C)
1074	1051	1080	1043	1075	ν(N=C)(CH_2_) + ν(C–C)
1131	1144	1144	1152	1147	δ(H–C–C–N)
1261	1292	1270	1276	1270	ν(N=C)(CS_2_) + δ(H–C–N–C) + β(H–C–C)
1367	1354	1350	1354	1366	δ(H–C–N–C) + β(H–C–H)(CH_2_)
1412	1416	1423	1447	1432	ν(N=C)(CS_2_) + β(H–C–H)(CH_2_)
1449	1462	1454	1470	1454	β(H–C–H)(CH_2_) + β(H–C–H)(CH_3_)
1474	1493	1490	1517	1504	ν(N=C)(CS_2_) + β(H–C–H)(CH_2_)

^a^ The scale factor of 0.97 was applied. ^b^ Abbreviations: δ: Torsion, ν: stretching, β: in-plane bending, γ: out-of-plane bending.

**Table 2:** Atomic percentages of various metal ionic species in electroplating wastewater samples.

Sample	Cr	Mn	Fe	Ni	Cu	Zn
“S1” (cyanide wastewater)	ND *	ND	342.95	703.85	84.69	2447.67
“S2” (after alkaline chlorination)	ND	2.38	468.28	667.66	77.06	2175.26

* ND: not detected.
